# The Fight-Or-Flight Response Is Associated with PBMC Expression Profiles Related to Immune Defence and Recovery in Swine

**DOI:** 10.1371/journal.pone.0120153

**Published:** 2015-03-20

**Authors:** Michael Oster, Mathias Scheel, Eduard Muráni, Siriluck Ponsuksili, Manuela Zebunke, Birger Puppe, Klaus Wimmers

**Affiliations:** 1 Leibniz Institute for Farm Animal Biology (FBN), Institute for Genome Biology, Wilhelm-Stahl-Allee 2, Dummerstorf, Germany; 2 Leibniz Institute for Farm Animal Biology (FBN), Institute for Behavioural Physiology, Wilhelm-Stahl-Allee 2, Dummerstorf, Germany; Faculdade de Medicina Dentária, Universidade do Porto, PORTUGAL

## Abstract

Defining phenotypes according to molecular features would promote the knowledge of functional traits like behaviour in both human and animal research. Beside physiological states or environmental factors, an innate predisposition of individual coping strategies was discussed, including the proactive and reactive pattern. According to backtest reactivity, animals assigned as high-resisting (proactive) and low-resisting (reactive) were immune challenged with tetanus toxoid in a time course experiment. Using the Affymetrix platform and qPCR, individual coping characteristics were reflected as gene expression signatures in porcine peripheral blood mononuclear cells (PBMC) at naïve state (day 0) and in response to the model antigen (day 14, day 28, and day 140). Further, the blood cell count was analysed at all stages. On the transcriptional level, processes acting on cell communication, vasculogenesis, and blood coagulation were highlighted in high-resisting animals at naïve state (day 0), temporarily blurred due to immune challenge (day 14) but subsequently restored and intensified (day 28). Notably, similar amounts of white and red blood cells, platelets and haematocrit between high-resisting and low-resisting samples suggest coping-specific expression patterns rather than alterations in blood cell distribution. Taken together, the gene expression patterns indicate that proactive pigs might favour molecular pathways enabling an effective strategy for defence and recovery. This corroborates the previously suggested belief, that proactive animals are prone to an increased number of injuries as an evolutionary inherited mechanism. In contrast to previous assumptions, coping-specific immunity in pigs lacks inherited shifts between cellular and humoral immune responses.

## Introduction

Phenotypic characteristics of functional traits like health, fertility, longevity, and behaviour have remained difficult to define as measurable molecular features despite extensive performance testing and novel insights through quantitative genetics [1,2]. In particular, substantial and reliable data on the impacts of genes on functional traits like behaviour, ‘disease resistance’, and ‘disease tolerance’ are scarce. However, interest in developing molecular signatures (e.g., gene expression profiles) is now growing exponentially, boosted by intentions to establish ethical husbandry and resource conservation in animal-source food production. Defining phenotypes according to molecular features would promote the knowledge of functional traits like behaviour in both human and animal research. Inter-individual variability provides some clues that will aid the identification of molecular signatures. For instance, individuals differ in the ways they cope with environmental challenges [3]. Coping styles have been shaped by evolution to form general adaptive response patterns. Two different coping styles have been distinguished [4]. The proactive, or active, pattern is characterized by a fight-or-flight response, high levels of aggression, and territorial control [5]. The reactive, or passive, pattern involves a conservation-withdrawal response, immobility, and low levels of aggression [6]. Interestingly, these distinct coping styles differ at the physiological and neuroendocrinological levels [7]. Animals with an active coping pattern have high sympathetic activity and moderate hypothalamic-pituitary-adrenal (HPA)-axis reactivity, probably resulting from preparation for action. In contrast, animals with a passive coping pattern have higher parasympathetic reactivity and high HPA axis activity. As the autonomic nervous system (i.e., the sympathetic branch) and the HPA axis communicate between the brain and the immune system, a differential reactivity of these systems may cause inter-individual differences in the immune response [8].

Coping styles have been studied in animals through behavioural tests. In particular, Hessing and collaborators applied the ‘backtest’, in which a pig is placed in a supine position for 60 seconds and monitored for struggling; they hypothesized that this simple method could detect coping styles in domestic pigs [9,10]. Subsequent applications of the backtest demonstrated individual consistency in behavioural responses over time [11,12] and linked neuroendocrine and physiological characteristics [13–15]. Indeed, reactivity to the backtest is believed to reflect basal molecular differences that correspond to immune features comprising both cellular and humoral immunity [15–17]. In particular, aggressive and resistant pigs (i.e., animals showing an active coping pattern) appeared to predominantly rely on cellular immune response, whereas non-aggressive and non-resistant pigs (i.e., animals showing a passive coping pattern) appeared to predominantly rely on humoral immune response [15,16].

However, evidence also suggests that primary behavioural differences and related physiological features between individuals appear particularly due to stressful situations [18]. In this context, the molecular responses producing phenotypes of interest can be investigated through immune challenges, which are, by nature, stressful. For example, vaccination with tetanus toxoid, used as model antigen, causes long-lasting cellular (Th1) and humoral (Th2) responses [19,20] via CD4^+^ MHC class II-restricted T helper cells [21]. The tetanus toxoid introduces a signal that initiates a coordinated program of gene expression in porcine peripheral blood mononuclear cells (PBMC) [22,23]. In a recent time-course experiment, tetanus toxoid vaccination produced phenotype-specific transcriptional differences in both immune features and metabolic pathways in animals divergent for lean growth performance [24]. These findings demonstrate that the stress of the immune challenge is associated with particular molecular signatures.

Here, we used temperament-dependent behaviour differences, resulting in either proactive (high-resisting) or reactive (low-resisting) coping patterns, to identify phenotypes related to immune response following tetanus toxoid vaccination. In this time-course experiment, molecular effects of an immune stimulation were investigated *in vivo*, focusing on piglets classified as either high or low-resisting according to latency, total duration, and frequency of struggling bouts in a backtest [12]. A recently-described whole genome microarray platform [25] was used to analyse gene expression in porcine PBMC before and after immune stimulation. We aimed to derive trait-associated molecular signatures applicable for selection according to (i) basal conditions regarding the experimental groups, and (ii) reactivity-dependent immune responses after vaccination. Changes in gene expression were quantified in porcine PBMC at 4 distinct time points.

## Materials and Methods

### Animals, performance tests, and vaccination

Animals were provided by the Leibniz Institute for Farm Animal Biology (FBN). Animal care, vaccination, and blood collection procedures followed the guidelines of the German Law of Animal Protection. The experimental protocol was approved by the Animal Care Committee of the Leibniz Institute of Farm Animal Biology and the State Mecklenburg-Western Pomerania (Landesamt für Landwirtschaft, Lebensmittelsicherheit und Fischerei; LALLF M-V/TSD/7221.3–2.1–020/09).

**Fig 1:**
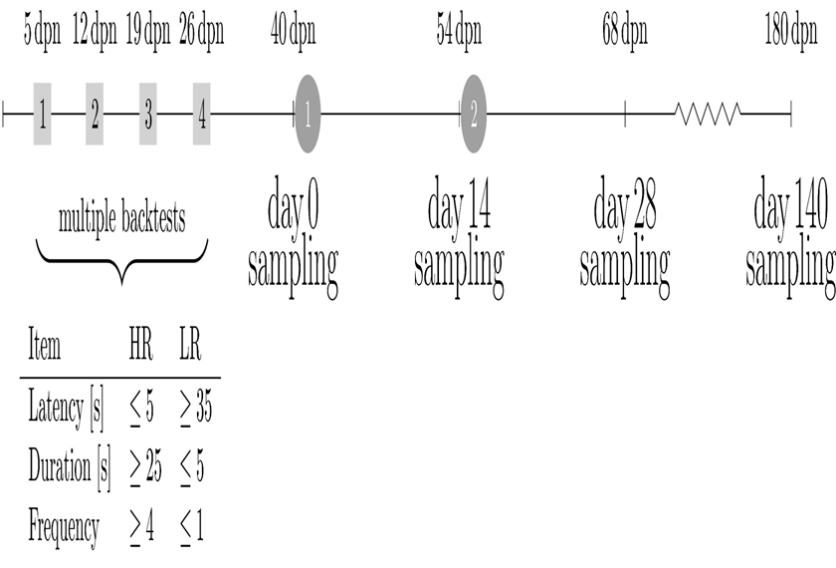
Experimental design. Pigs were subjected to the backtest at 5 dpn, 12 dpn, 19 dpn, and 26 dpn as indicated by the grey rectangles, resulting in 12 individual parameters considered for classification into HR and LR [12]. The grey circles indicate 1^st^ and 2^nd^ (booster) tetanus toxoid vaccinations shortly after day 0 and day 14 sampling points. Blood (n = 48) was collected at four sampling points: in naïve state (day 0), 14 days after 1^st^ vaccination (day 14), 14 days after 2^nd^ vaccination (day 28), and at slaughter (day 140); dpn = days post-natum.

The experimental design is detailed in Fig. 1. German Landrace piglets were part of a larger study and were subjected to a backtest as described [12]. In brief, the backtest was performed on 3,555 piglets (1,759 males and 1,796 females derived from 223 sows and 42 boars) at four time points [5 days post-natum (dpn), 12 dpn, 19 dpn, 26 dpn]. Each backtest lasted 60 seconds. Several criteria were used to classify the investigated piglets as high-resisting (HR), doubtful (D), or low-resisting (LR) animals [13]: (i) latency, or time until first response (HR: ≤ 5s; LR: ≥ 35s); (ii) total duration, or cumulative time interval of responses (HR: ≥ 25s; LR ≤ 5s); and (iii) frequency of struggling bouts (HR: ≥ 4; LR ≤ 1). Regarding to the four measurement periods, each of the individual 12 parameters was considered for classification into HR, D, or LR [12]. In total, 417 piglets were identified as HR animals (~12%) and 784 piglets were identified as LR animals (~22%).

In general, weaning appears to be a stressful period in life during which an impaired immune responsiveness occurs in pigs [26,27]. Piglets were weaned at 28 dpn. Hence, to eliminate eventual effects, the immune challenge started at approximately 40 dpn when a subset of the classified five-week-old piglets (HR: 145; LR: 107; litters obtained in 2009 and 2010) was subcutaneously vaccinated (day 0) with one dose (1 mL) of tetanus vaccine, comprising tetanus toxoid and aluminium hydroxide as adjuvant (Equilis Tetanus-Vaccine, Intervet, Unterschleißheim, Germany). After two weeks, a booster vaccination was given (day 14). Note, males were castrated at 4 dpn.

### Blood samples

Blood samples were collected from jugular veins into EDTA-treated tubes immediately before the first (day 0) and second vaccinations (day 14) as well as two weeks after the booster vaccination (day 28). Blood parameters (leucocytes, lymphocytes, monocytes, neutrophils, eosinophils, basophils, platelets, erythrocytes, and haematocrit) were analysed at day 0, day 14, and day 28 (ABX Pentra 60, HORIBA ABX SAS, Montpellier, France). Additionally, trunk blood was collected at slaughtering (day 140), when pigs were weighed and slaughtered by electronarcosis followed by exsanguination in the experimental slaughterhouse of FBN. Trunk blood was supplemented with EDTA and stored on ice.

### RNA isolation, target preparation, and hybridization

For expression analysis a subset of animals was selected aiming for a balanced design in terms of gender, batch and ancestry. Accordingly, twelve castrated males and twelve females per coping group were selected for subsequent analyses, producing 48 individual samples per sampling time point. The PBMCs were isolated from 5 mL blood by centrifugation on a Histopaque density gradient (Sigma-Aldrich, Taufkirchen, Germany), then stored at -80°C. Total RNA was isolated using Qiazol reagent per manufacturer’s directions (Qiagen, Hilden, Germany). Quantification and purification were performed as previously described [24]. All RNA was stored at -80°C until downstream analyses were performed. For the microarray experiments, individual samples (n = 192) were hybridized on genome-wide *snowball* arrays (Affymetrix, Santa Clara, CA, USA), a platform invented for genome-wide analyses of the pig transcriptome [25]. Processing was performed as previously described [24]. Raw data have been deposited in a MIAME-compliant database, the National Center for Biotechnology Information Gene Expression Omnibus (www.ncbi.nlm.nih.gov/geo) (accession numbers: GSE55418).

### Data analyses

In total, 188 of 192 arrays passed the appropriate quality control criteria as proposed by Kauffmann *et al*. [28]. Data were RMA-normalized (Log2). To improve statistical power [29], inappropriate probe-sets were excluded from further analyses, such as probe-sets with a small logarithmized mean (m<2.5) among all analysed arrays as well as probe-sets with a small standard deviation (SD<0.22) among all arrays included in the analysis. It was assumed that transcriptional differences resulted from genetic factors (paternal effects), time-dependent response to vaccination, and different phenotypes of temperament. Therefore, relative mRNA differences (p≤0.01) including individual and combined effects of coping group, sire (partially confounded to coping group), time, and batch were analysed using a mixed model [V_ijkl_ = μ + coping group_i_ + time_j_ + batch_k_ + sire_l_(coping group_i_) + (coping group x time)_ij_ + e_ijkl_]. The mixed model was combined with a repeated statement for the time component specified as heterogeneous covariance structure (SAS version 9.3; SAS Institute, Cary, NC, USA). Sampling time points were weighted equally while comparing phenotypes divergent in temperament. Due to multiple testing, p-values were converted to a set of q-values [30]. The level of significance was set at q≤0.25.

### Pathway analyses

Annotation data for Affymetrix *snowball* arrays were obtained from the developers [25]. Gene lists obtained from the PBMC microarray analyses were evaluated with ‘Ingenuity Pathway Analysis’ (IPA, Ingenuity Systems, Redwood City, CA, USA). The significance of association between dataset and pathway/biofunction analyses was calculated (p≤0.05). The top 10 pathways showing the lowest p-values were considered for further analyses. To identify relevant IPA-biofunctions, the *z*-score was used to discriminate between increased (*z* > 2; HR > LR) or decreased (*z* < -2; HR < LR) functional themes.

### Quantitative real-time PCR

Selected reference genes were characterized by small standard deviation between coping groups (SD<0.1). Further, their expression values (log2) ranged approximately at the level of the target genes. Total transcript levels of selected target (*CD69*, *GNAZ*, *ITGA2B*) and reference genes (*IQGAP1*, *TSC22D2*) were quantified by real-time qPCR (S1 Table). Individual PBMC mRNA samples (n = 44 per sampling day) were analysed in duplicate on a LightCycler 480 system using LightCycler 480 SYBR Green I Master (Roche, Mannheim, Germany). Data were factorial normalized. The statistical analysis included individual and combined effects of coping group, sire (confounded to coping group), time, and batch (SAS version 9.3; SAS Institute, Cary, NC, USA). The level of significance was set at p≤0.05.

## Results

The *snowball* microarray covers 47,845 probe-sets corresponding to 17,964 annotated genes. After filtering as described above, 27,033 probe-sets (~57%) remained for analysis. These probe-sets corresponded to 11,620 annotated genes.

### Transcriptional responses in animals divergent in temperament

The comparison of porcine PBMC derived from HR and LR samples revealed a number of probe-sets differing in their mRNA abundance by both coping group and sampling points (S2 Table).

To study expression patterns at naïve state and due to immune challenges in animals divergent in coping style, pathways that showed effects mediated by ‘coping group’ were examined in detail at all sampling points (Table 1, S3 Table). In general, the selected top 10 canonical pathways reflected consistent time-dependent patterns. Analysis of transcriptional differences suggested molecular routes differing between HR and LR samples both at day 0 and day 28 (‘α-Adrenergic Signaling’, ‘G Beta Gamma Signaling’, ‘Thrombin Signaling’, ‘Integrin Signaling’, ‘Protein Kinase A Signaling’, ‘RhoGDI Signaling’), as well as at day 140 (‘P2Y Purigenic Receptor Signaling Pathway’, ‘IL-8 Signaling’, ‘Gap Junction Signaling’, ‘CXCR4 Signaling’). Among those pathways, only ‘CXCR4 Signaling’ was also altered at day 14. A complete list of the altered transcripts associated with the displayed canonical pathways is provided in S3 Table.

**Table 1:** Top 10 Ingenuity pathways of transcripts with higher and lower expression between HR and LR PBMC samples at four time points (day 0, day 14, day 28, and day 140).

Canonical pathway	Sampling day	p-value	Number of involved genes
P2Y Purigenic Receptor Signaling Pathway	day 0	8.51E-04	9
	day 14	2.44E-01	1
	day 28	1.51E-05	9
	day 140	2.24E-02	4
IL-8 Signaling	day 0	1.55E-03	11
	day 14	6.76E-02	2
	day 28	8.32E-05	10
	day 140	2.40E-02	5
Gap Junction Signaling	day 0	4.57E-03	9
	day 14	3.01E-01	1
	day 28	1.05E-04	9
	day 140	1.20E-02	5
α-Adrenergic Signaling	day 0	4.07E-04	8
	day 14	1.83E-01	1
	day 28	6.31E-04	6
	day 140	1.79E-01	2
G Beta Gamma Signaling	day 0	7.94E-05	9
	day 14	1.85E-01	1
	day 28	6.76E-04	6
	day 140	1.82E-01	2
Thrombin Signaling	day 0	5.37E-04	12
	day 14	7.08E-02	2
	day 28	9.77E-05	10
	day 140	8.71E-02	4
CXCR4 Signaling	day 0	1.15E-03	10
	day 14	4.79E-02	2
	day 28	4.90E-04	8
	day 140	4.47E-02	4
Integrin Signaling	day 0	1.23E-05	15
	day 14	3.64E-01	1
	day 28	4.27E-06	12
	day 140	2.47E-01	3
Protein Kinase A Signaling	day 0	1.05E-05	22
	day 14	5.83E-01	1
	day 28	2.00E-03	12
	day 140	2.37E-01	5
RhoGDI Signaling	day 0	5.01E-05	13
	day 14	5.89E-02	2
	day 28	4.07E-05	10
	day 140	-	-

Metadata of involved genes were displayed in S3 Table. HR—High resisting; LR—Low resisting.

To gain insight into functional and structural components, genes with altered mRNA abundances were assigned to IPA-biofunctions. Here, the *z*-score was used (z > 2 or z < -2) to identify functional themes with respect to stage-specific alterations. The analyses revealed transcriptional differences at day 0 and day 28 only. Biofunctions that appeared to be altered between coping groups at both day 0 and day 28 are listed in Table 2. Moreover, biofunctions revealing a molecular signature specifically for either day 0 or day 28 are shown in Tables 3 and 4, respectively. In general, the addressed biofunctions corresponded to themes like ‘Cell-to-cell Signaling and Interaction’, ‘Cellular Assembly and Organization’, ‘Haematological System Development and Function’, ‘Immune Cell Trafficking’, ‘Inflammatory Response’, ‘Tissue Development’, and ‘Tissue Morphology’. A complete list of the altered transcripts associated with the displayed biofunctions at day 0 and day 28 is provided in S4 Table.

**Table 2:** Common Ingenuity biofunctions of transcripts with higher and lower expression between HR and LR PBMC samples at day 0 and day 28.

Biofunction	mRNA abundance	p-value	Number of involved genes
adhesion of endothelial cells	HR > LR; day 0	9.37E-03	7
	HR > LR; day 28	2.63E-03	6
aggregation of blood platelets	HR > LR; day 0	1.65E-04	12
	HR > LR; day 28	1.09E-07	13
bleeding time	HR < LR; day 0	3.58E-05	7
	HR < LR; day 28	6.16E-08	8
quantity of lymphatic system cells	HR < LR; day 0	3.88E-03	14
	HR < LR; day 28	1.09E-02	9

Metadata of involved genes were displayed in S4 Table. HR—High resisting; LR—Low resisting.

**Table 3:** Specific Ingenuity biofunctions of transcripts with higher and lower expression between HR and LR PRMC samples at day 0.

Biofunction	mRNA abundance	p-value	Number of involved genes
adhesion of vascular endothelial cells	HR > LR; day 0	1.29E-02	5
quantity of blood platelets	HR > LR; day 0	9.55E-04	9
vasculogenesis	HR > LR; day 0	9.77E-03	27
quantity of megakaryocytes	HR < LR; day 0	2.30E-03	5
senescence of cells	HR < LR; day 0	1.14E-02	10

Metadata of involved genes are displayed in S4 Table. HR—High resisting; LR—Low resisting.

**Table 4:** Specific Ingenuity biofunctions of transcripts with higher and lower expression between HR and LR PBMC samples at day 28.

Biofunction	mRNA abundance	p-value	Number of involved genes
adhesion of blood cells	HR > LR; day 28	1.30E-04	15
adhesion of granulocytes	HR > LR; day 28	5.77E-03	5
attachment of cells	HR > LR; day 28	3.82E-04	7
binding of blood cells	HR > LR; day 28	2.77E-03	9
binding of blood platelets	HR > LR; day 28	1.88E-05	6
binding of cells	HR > LR; day 28	1.14E-05	19
cell movement	HR > LR; day 28	8.93E-08	55
cell movement of leukocytes	HR > LR; day 28	2.31E-03	21
cell movement of mononuclear leukocytes	HR > LR; day 28	4.01E-03	14
cell movement of myeloid cells	HR > LR; day 28	5.40E-03	15
cell movement of neutrophils	HR > LR; day 28	1.18E-02	9
cell viability of blood cells	HR > LR; day 28	1.09E-02	9
cell viability of hematopoietic progenitor cells	HR > LR; day 28	2.79E-03	5
chemotaxis of cells	HR > LR; day 28	2.51E-04	17
chemotaxis of leukocytes	HR > LR; day 28	2.61E-03	12
chemotaxis of myeloid cells	HR > LR; day 28	1.16E-02	9
engulfment of cells	HR > LR; day 28	1.20E-03	12
homing of cells	HR > LR; day 28	1.85E-04	18
homing of leukocytes	HR > LR; day 28	1.49E-03	13
invasion of cells	HR > LR; day 28	3.49E-04	20
leukocyte migration	HR > LR; day 28	2.22E-04	26
MAPKKK cascade	HR > LR; day 28	1.75E-03	8
microtubule dynamics	HR > LR; day 28	4.47E-06	27
migration of cells	HR > LR; day 28	4.19E-07	50
morphology of cells	HR < LR; day 28	8.06E-04	35
organization of cytoskeleton	HR > LR; day 28	4.26E-07	34

Metadata of involved genes are displayed in S4 Table. HR—High resisting; LR—Low resisting.

### Cluster analyses

**Fig 2:**
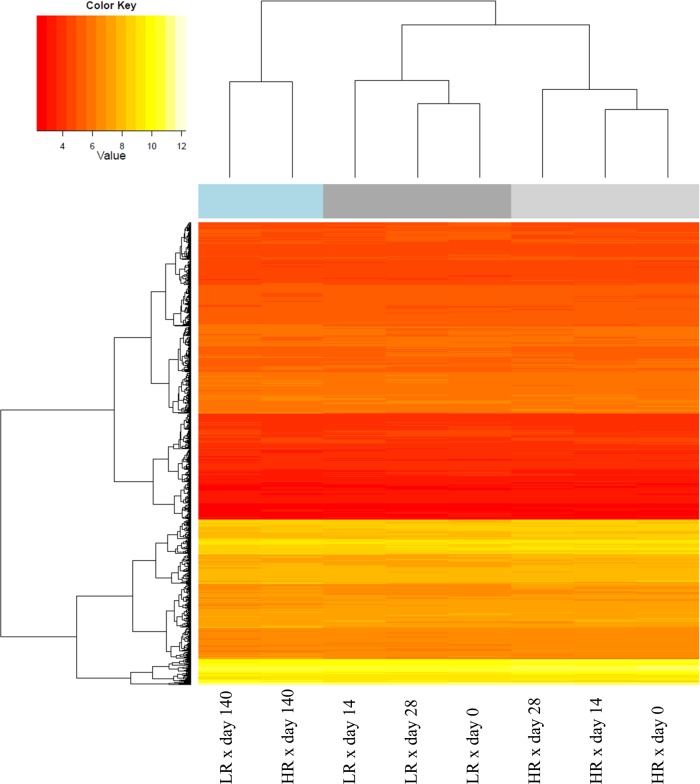
Heatmap displaying probe-sets with significantly altered mRNA abundances. Effects mediated by coping group appeared to dominate early sampling points (day 0, day 14, day 28). Later, age-specific effects were more pronounced as visualized by young adult subgroups (day 140). Columns = variance component coping group x time; Rows = transcripts showing altered mRNA abundances between HR and LR on at least one time point; HR—High resisting; LR—Low resisting.

The impact of coping group and time on gene expression was visualized by hierarchical clustering, accounting for significantly altered probe-sets of all investigated subgroups (Fig. 2). Clustering revealed two superior clusters, where coping group appeared to dominate early sampling points (HR x day 0, HR x day 14, HR x day 28, and LR x day 0, LR x day 28, LR x day 14, respectively). The remaining cluster consisted of the subgroups HR x day 140 and LR x day 140, indicating a minor impact of coping group on gene expression at slaughter age. Notably, the final slaughter weight was similar between experimental groups (HR: 110.5 ± 7.6 kg and LR: 112.0 ± 7.6 kg, respectively).

### Alterations in mRNA abundances of selected transcripts

**Fig 3:**
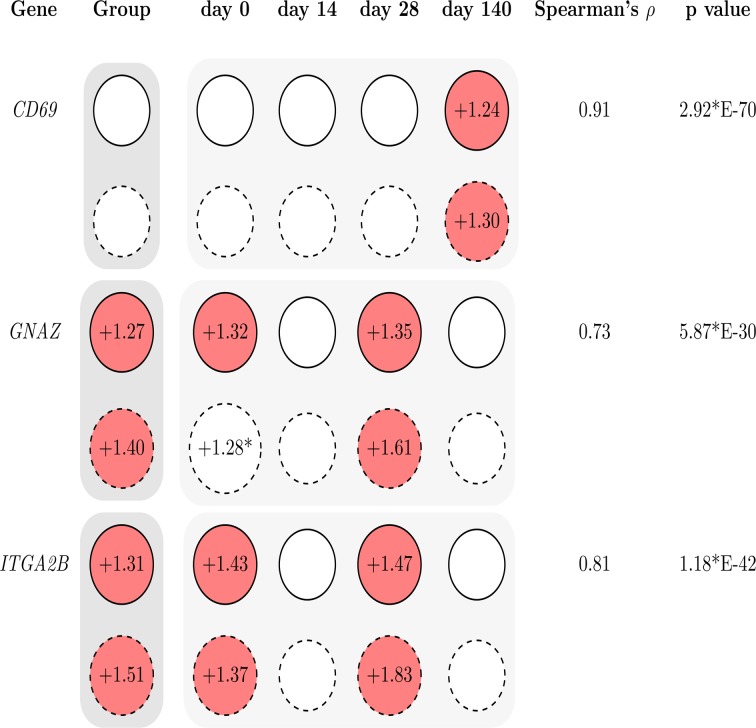
Comparison of microarray and quantitative PCR (qPCR) results for selected transcripts (*CD69*, *GNAZ*, *ITGA2B*) to verify microarray data. Values were calculated by factorial normalisation on *IQGAP1* and *TSC22D2* expression values. Fold-changes displayed in red circles indicate significant differences in mRNA abundances between HR and LR at either microarray (solid lined circles) or qPCR data (dashed lined circles). Positive values display increased mRNA abundances in HR (HR > LR). Correlation of normalized expression values was calculated by Spearman (n = 176). * p = 0.06.

Both microarray and qRT-PCR analyses were correlated to verify differences in mRNA abundance of genes encoding cell surface receptors (*CD69*, *GNAZ*, *ITGA2B*) between coping groups (Fig. 3). The transcripts were analysed at multiple sampling points. The fold-changes revealed a reliable dimension. Between microarray and qPCR data the correlation coefficients were highly significant and ranged between 0.73 and 0.91. Taken together, the qPCR analyses indicate reproducibility of the microarray analysis.

### Similar cell blood count in HR and LR samples

At day 0, day 14, and day 28, HR and LR samples were unaltered regarding their total leucocyte number, lymphocytes, monocytes, neutrophils, eosinophils, basophils, platelet number, erythrocyte number, and haematocrit (S1 Fig.).

## Discussion

Both genetic [11,31,32] and environmental factors [33] appear to contribute to determining coping characteristics in pigs. In particular, extrinsic and intrinsic cues are perceived by the brain, which, in turn, orchestrates appropriate behavioural responses. In this context, due to the linkage between brain and immune system, both efferent and afferent signals enact sensor and effector functions of the immune system [34–36]. Expanding upon and consistent with *in vivo* situations, the current study investigated transcriptional patterns in peripheral immunocompetent cells obtained from pigs divergent for temperament. The individual coping characteristics were reflected as transcriptional differences at naïve state and in response to the immune challenge. Notably, the unchanged absolute and relative amounts of white blood cells, red blood cells, thrombocytes, and the haematocrit reflect the temperament-specific character of the observed mRNA alterations.

### Re-establishing transcriptional differences following the immune challenge in juveniles

Our analysis revealed higher basal gene expression of transcripts associated with cell communication, vasculogenesis, pro-inflammation, and wound healing in HR animals at day 0. Interestingly, the observed differences at naïve stage disappeared at day 14, suggesting that the acute response to the model toxoid is similar in terms of extent and type in both HR and LR animals. Hence, responses due to acute immune challenges do likely dominate subtle coping-specific differences. In contrast, the PBMC signature was characterized by the re-establishment of the naïve patterns at day 28, promoting molecular routes related to vasculogenesis and pro-inflammation. Moreover, the assigned IPA biofunctions highlighted coping specificities for predominantly haematological processes, including cell communication, vasculogenesis, and blood coagulation.

Taken together, our analysis reveals heterogeneous molecular signatures between HR and LR samples, particularly for molecular pathways associated with vasculogenesis and pro-inflammation. Therefore, the temperament-dependent gene expression might reflect differences in the platelet—leukocyte interaction. Indeed, the cross-talk between blood cells has a bidirectional character, highlighting lymphocyte engagement in primary steps of blood coagulation, as reviewed elsewhere [37,38].

Since the divergent expression patterns were more pronounced at day 28, these findings support the growing evidence that challenging situations promote the unravelling of the subtle inherited differences in behaviour-related molecular features [18]. The observed expression patterns suggest that HR animals inherited vigilant basal immune functions that manifest independently of any wounding or injuries. Indeed, animals that are prone to experience stressful events do likely have to deal with an increased number of injuries. Thus, as suggested previously, HR animals might favour an evolutionary inherited mechanism that interrelate a physically active behaviour with the immune system [39]. However, it is of particular scientific interest whether a potential ‘improved’ immune status was developed at the expense of a predisposition for adverse immunological outcomes [40]. Superficially, this would dilate the hypothesis concerning a trade-off between immunity and stress response [41] to a trade-off between immunity and temperament. However, this remains to be scientifically investigated.

### Temperament-specific transcriptional differences in young adult animals

At day 140 the analyses revealed stage-specific transcriptional responses, accompanied by a lack of relevant IPA-biofunctions. Hence, both age and coping group influenced the immune response in growing pigs, a finding that corresponds to previous observations made when baseline immune measures were analysed [17,42]. In contrast, no differences in immune properties were found between HR and LR pigs at the age of 12 months [43]. These conflicting observations account, at least in part, for the limited prior success in the discovery of molecular features related to coping groups in young adult animals. Despite reports that coping styles in farm animals such as cattle are stable over years [44], it is conceivable that subtle unnoticed differences in temperament traits become more pronounced by age [45,46]. Further, predicted personality traits such as boldness and docility are considered to be age-related in non-human mammals [47]. Consequently, temperament-specific molecular features such as immune responses might also be modified with age.

### Coping style and immunity

HR and LR pigs are reportedly distinguishable by their favoured immune response, preferentially using either cellular or humoral immunity [15,16]. In contrast, HR pigs exhibited an increased lymphocyte proliferation following DNP-KLH-stimulation [17]. However, in our study no distinct shift between Th1 and Th2 immune responses was observed. Further, we found that mRNA abundances of genes associated with α-adrenergic signaling were mostly decreased in HR animals at both day 0 and day 28. Because α-adrenergic signaling is known to mediate parts of the diverse biological effects of the sympathetic branch of the autonomic nervous system, those findings seems contradictory to the established theories. Thus, the polarization into either HR or LR animals featuring (i) an emphasis on cellular immune response, moderate HPA reactivity, and sympathetic activation in HR samples versus (ii) an emphasis on humoral immune response, HPA activity, and parasymthatetic reactivity in LR samples may be rather incomplete. Taken together, the general model relating coping style, autonomous nervous system, and immune properties may lack additional genetic and environmental interactions.

### Individual variation in modern breeding systems

Currently, farm animal breeding aims to reduce individual variation by strong selection criteria corresponding to various traits. As a side effect, these intentions may have narrowed the spectrum of principles underlying genotypic variation and phenotypic plasticity [7]. Indeed, in rats the reduction in individual variation due to inbreeding contributes to an altered frequency distribution of behavioural traits, creating a paucity of individuals exhibiting extreme responses [7]. Accordingly, due to accelerated animal breeding and the need to adapt to modern housing systems [48,49] (e.g., decreased space, high temperature, high levels of noxious gases, high stocking density, missing wallow, increased bacterial load) it is very likely that similar results will be found in domesticated pig breeds. However, our data indicate that in terms of coping behaviour a great variation exists among pigs.

### Conclusion

In this study, the coping groups high-resisting (HR) and low-resisting (LR) were successfully discriminated according to their molecular features related to cell communication, vasculogenesis, and blood coagulation, although the tetanus toxoid vaccination blurred transcriptional differences between coping groups temporarily (day 14). However, the distinct expression signatures observed at naïve state were even intensified at day 28, as reflected by transcripts associated with platelet—leukocyte interaction. Thus, at juvenile stages it appears that HR pigs might favour molecular pathways enabling an effective strategy for defence and recovery. In contrast to previous assumptions, coping-specific immunity in pigs lacks inherited shifts between Th1 and Th2 immune responses. Notably, the similar amounts of white and red blood cells, platelets and haematocrit between HR and LR samples suggest coping-specific expression patterns rather than alterations in blood cell distribution. Thus, blood could be a suitable tissue to obtain molecular markers for distinct coping styles that might be used to drive genetic selection decisions, to optimize animal management, and for in-depth, molecular phenotyping of animals for further research of the relationships of behaviour, immune traits and production traits.

## Supporting Information

S1 Fig Blood parameters between HR (unfilled boxes) and LR (grey boxes) PBMC samples at day 0, day14, and day 28. (TIF)

S1 Table Primers used in qPCR to verify microarray results. (XLS)

S2 Table Transcripts differing in mRNA abundance between HR and LR PBMC samples. (XLS)

S3 Table Transcripts differing in mRNA abundance between HR and LR PBMC samples are enriched in certain canonical pathways at day 0, day 14, day 28, and day 140. (XLS)

S4 Table Transcripts differing in mRNA abundance between HR and LR PBMC samples are enriched in certain biofunctions at day 0 and day 28. (XLS)
