# Supplementary figures and images for: The Fight-Or-Flight Response Is Associated with PBMC Expression Profiles Related to Immune Defence and Recovery in Swine
Source: PLoS One. 2015 Mar 20;10(3):e0120153. doi: 10.1371/journal.pone.0120153 (PMC4368799; doi:10.1371/journal.pone.0120153)

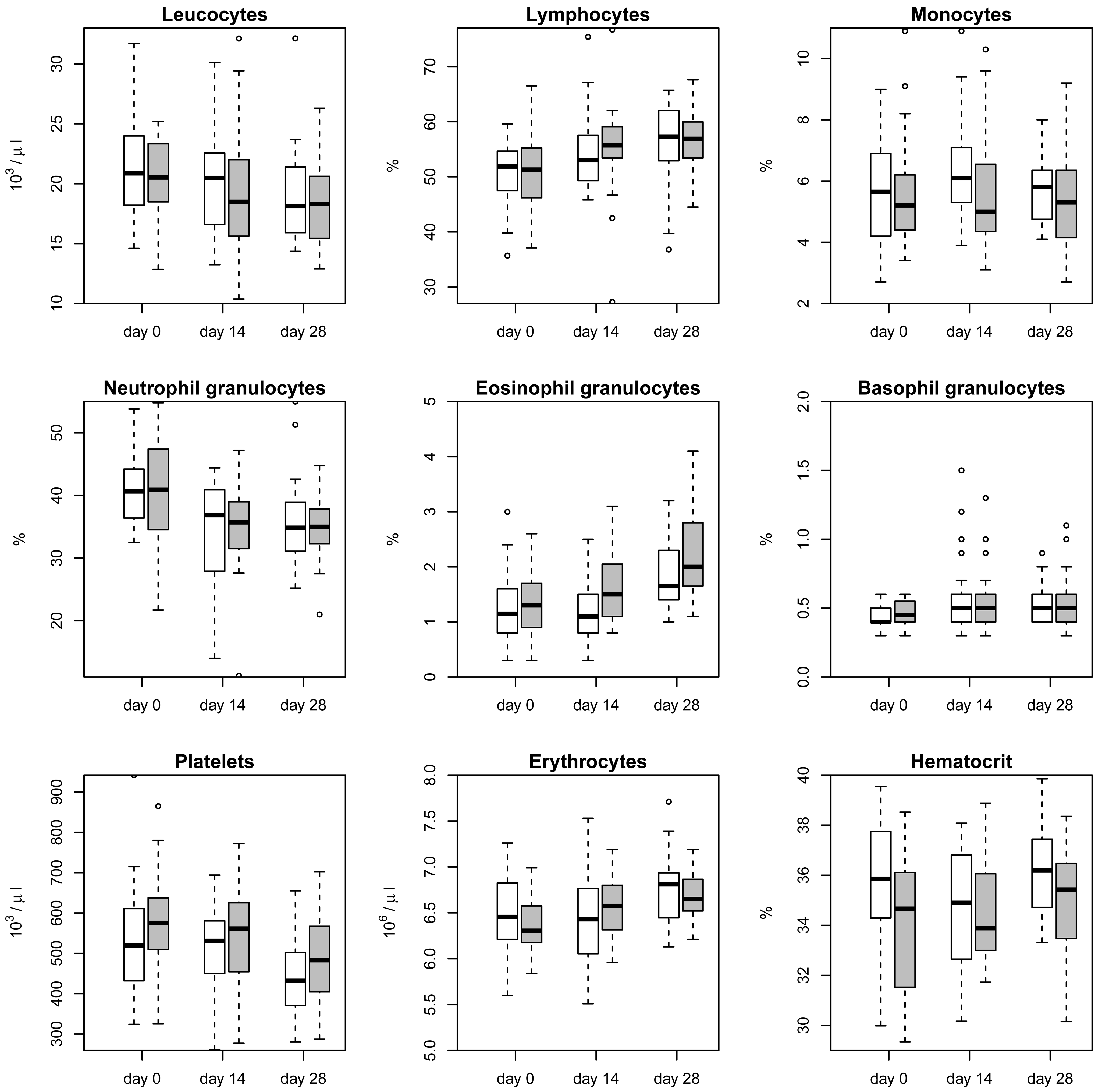

Supplement: S1 Fig — (TIF) [file pone.0120153.s001.tif]
